# Patient Access, Unmet Medical Need, Expected Benefits, and Concerns Related to the Utilisation of Biosimilars in Eastern European Countries: A Survey of Experts

**DOI:** 10.1155/2018/9597362

**Published:** 2018-01-10

**Authors:** Andras Inotai, Marcell Csanadi, Guenka Petrova, Maria Dimitrova, Tomasz Bochenek, Tomas Tesar, Kristina York, Leos Fuksa, Alexander Kostyuk, Laszlo Lorenzovici, Vitaly Omelyanovskiy, Katalin Egyed, Zoltan Kalo

**Affiliations:** ^1^Syreon Research Institute, Budapest, Hungary; ^2^Department of Health Policy and Health Economics, Eötvös Loránd University (ELTE), Budapest, Hungary; ^3^Department of Pharmaceutics and Central Clinical Pharmacy, University of Pécs, Pécs, Hungary; ^4^Faculty of Pharmacy, Medical University-Sofia, Sofia, Bulgaria; ^5^Department of Drug Management, Faculty of Health Sciences, Jagiellonian University Medical College, Krakow, Poland; ^6^Department of Organisation and Management in Pharmacy, Faculty of Pharmacy, Comenius University, Bratislava, Slovakia; ^7^Management Center Innsbruck, Innsbruck, Austria; ^8^Department of Social and Clinical Pharmacy, Faculty of Pharmacy, Charles University in Prague, Hradec Králové, Czech Republic; ^9^Kazakh Agency for Health Technology Assessment, Astana, Kazakhstan; ^10^Syreon Research Romania, Tirgu Mures, Romania; ^11^Faculty of Technical and Human Sciences, Sapientia University, Tirgu Mures, Romania; ^12^Center of Healthcare Quality Assessment and Control, Ministry of Health of the Russian Federation, Moscow, Russia; ^13^Egis Pharmaceuticals, Budapest, Hungary

## Abstract

This policy research aims to map patient access barriers to biologic treatments, to explore how increased uptake of biosimilars may lower these hurdles and to identify factors limiting the increased utilisation of biosimilars. A policy survey was developed to review these questions in 10 Central and Eastern European (CEE) and Commonwealth of Independent States (CIS) countries. Two experts (one public and one private sector representative) from each country completed the survey. Questions were related to patient access, purchasing, clinical practice, and real-world data collection on both original biologics and biosimilars. Restrictions on the number of patients that can be treated and related waiting lists were reported as key patient access barriers. According to respondents, for both clinicians and payers the primary benefit of switching patients to biosimilars would be to treat more patients. However, concerns with therapeutic equivalence and fear of immunogenicity may reduce utilisation of biosimilars. Similar limitations in patient access to both original biologics and biosimilars raise concerns about the appropriateness and success of current biosimilar policies in CEE and CIS countries. The conceptual framework for additional real-world data collection exists in all countries which may provide a basis for future risk-management activities including vigorous pharmacovigilance data collection.

## 1. Introduction

All European countries are facing the challenge of how to cover and reimburse high priced patent-protected medicines such as biologic treatments from their limited health care budget. Despite a greater unmet medical need due to shorter life expectancy and worse overall health status of the population compared to higher income Western European countries, financial constraints are greater in countries of Central and Eastern Europe (CEE) and Commonwealth of Independent States (CIS) [1]. However, due to external price referencing, prices of newly developed medicines are often adjusted to the price prevailing in higher income countries, and launch sequence strategies are carefully designed by manufacturers to minimize the price erosion in countries with the greatest market potential [2–4]. In contrast, the cost of health services is much lower in CEE and CIS countries; therefore, a price similar to that in higher income countries cannot be justified based on cost savings from avoided medical events or hospitalisation [5]. As a consequence, the majority of new medicines, which may be cost-effective in higher income European countries, are not necessarily cost-effective in CEE and CIS countries [6]. Furthermore, lower income countries are not able to pay as much for improvements in mortality and morbidity. Still, as there is a strong political pressure to provide access to and reimbursement for such medicines, payers in lower income countries apply special cost-containment measures to facilitate the sustainability of health care financing. Confidential price reductions or discounts may be a reasonable method to tackle the narrow international price corridor of pharmaceuticals due to external price referencing: however, as several higher income European countries also benefit from confidential pricing agreements, other tools are also necessary. Further frequently applied—sometimes contradictory—methods to control access and pharmaceutical spending in countries with severe economic constraints include the following: delayed reimbursement of biologic medicines; restricted volume of patients on reimbursed biologics; limited treatment duration; introduction of waiting lists; and narrowed reimbursed indication compared to the registered indication specified in the Summary of Product Characteristics [7–10]. The existence of these barriers has been based on anecdotal evidence in the CEE and CIS region: hence, more field research, such as expert interviews focusing on these issues, is required to formulate international and national policies to reduce inequities in patient access and health.

The policy rationale for allowing biosimilar entry is analogous to that for generic entry following patent expiry for small molecules. Once patents expire, competing copies or near copies are permitted to enter the market. This competition will lead to lower prices, but generally as a function of the number of entrants. However, the costs of developing a biosimilar are much higher than those for a small molecule follow-on medicine. For the former, regulators require noninferiority head-to-head clinical trials versus the originator biologic medicine. For the latter, they require only bioequivalence assessed using laboratory methods. Generally, market prices decline with the number of entrants and in case of generic competition are often 10–20% of the originator's price. Markets for biologics with competing biosimilars may have fewer entrants and less price reduction, meaning that access will be more limited. Outcomes-based agreement that allow differential pricing in CEE and CIS countries could help to overcome this effect [11].

A large number of original biologic medicines is about to lose patent protection in the near future [12]. In fact, the so-called “patent cliff” for biologics has already begun since the top ten selling products in 2011 (worth 37.6% of the total biologics market value—almost 60 billion USD in global sales as of 2011) will lose their market exclusivity between 2012 and 2019 [13]. High-quality biosimilars authorised by reputable regulatory agencies such as the European Medicines Agency (EMA) or the Food and Drug Administration (FDA) offer therapeutic alternatives to original biologics at reduced price [14, 15]. However, the clinical and economic benefit of such biosimilars is not only to generate savings for health care budgets but also to improve patient access to reimbursed biologic products at these lower prices [16].

Compared to small molecule products, biologic therapies with large molecule weight and complex chemical structure cannot be reproduced in a completely identical form [17]. Therefore, confirmation of biosimilarity requires a more comprehensive evaluation and comparison of the quality, safety, and efficacy of the similar biologic product and its originator [18]. Despite the application of rigorous criteria, a potential concern about biosimilars is the extrapolation of clinical data required for registration to all indications of the original product [19].

A systematic literature review found that biosimilar immunogenicity differs among active compounds suggesting that immunogenicity of anti-drug antibodies should be an important consideration in the treatment decision-making process such as switching [20]. Some opinion leaders argue that switching from an original biologic medicine to a biosimilar may induce increased immunogenic reactions [21, 22]. Recent systematic reviews showed, however, that switching patients from the original chronic biologic therapy to a biosimilar alternative was not associated with increased risk of adverse reactions or loss of efficacy [23, 24]. Still, even with these recent findings, the utilisation of biosimilars, especially for patients on maintenance original biologic treatment, is not an obvious alternative for many physicians and payers [25–28].

The objectives of our policy research were to

1. map the barriers of patient access to biologic monoclonal antibodies in CEE and CIS countries,
2. explore how increased uptake of biosimilar monoclonal antibodies may lower these hurdles,
3. identify factors limiting the increased utilisation of biosimilar monoclonal antibodies.

## 2. Materials and Methods

A standardized English language survey was developed for collecting feedback from policy experts in 10 CEE and CIS countries, including Bulgaria, Czech Republic, Hungary, Kazakhstan, Latvia, Lithuania, Poland, Romania, Russia, and Slovakia. In each country, one expert from the public sector and one from the private sector completed the survey (see Table 1). Experts were selected based on their in-depth knowledge on biosimilar pharmaceutical policies in their own countries. Willingness to participate in the study was confirmed by each expert prior to the distribution of the survey, resulting in 100% overall response rate. The survey was distributed in an Excel spreadsheet format. Answers were received between March and September of 2015.

Survey questions are summarized in Table 2. To gain full understanding on the policy environment of each country, survey topics included a wide spectrum of questions on registration, pricing, reimbursement, patient access to reimbursed biologic treatment, and clinical practice such as switching to biosimilar medicines, purchasing practices, and collection of real-world data of original biologics and biosimilars. Beyond the general national biosimilar policy context, we specifically focused on monoclonal antibodies for the following reasons: (1) they have more complex protein structure compared to first-generation biosimilars such as erythropoietins, (2) they are associated with higher annual treatment cost and budget impact [29], (3) the patents of several monoclonal antibodies have expired recently or will expire soon, and (4) they are used for longer-term treatment in chronic conditions. Specific survey questions were focused on three therapies: infliximab, trastuzumab, and rituximab. However, at the time of this study period, no biosimilar alternatives were available for trastuzumab and rituximab. Thus, respondents were asked to express their future expectations based on their current knowledge, previous experiences, and individual perceptions. Terms used in the survey were explained in a glossary to minimize misunderstanding.

Iterative development of the survey tool covered preliminary interviews, field testing with local experts, and finalising it based on their comments before release. The final survey consisted of 4 spreadsheets (one for biologics in general and one each for infliximab, trastuzumab, and rituximab, resp.). Answer options were given in the survey, but respondents had a chance to provide additional clarification by using an “other (please specify)” option after each question. In addition, respondents could provide additional explanation or references. Responses by the two experts from each country were reconciled and in case of inconsistency disagreements were solved via e-mail queries.

## 3. Results

Survey results are presented according to the study objectives, reflecting some key areas of improving patient access to biosimilar products.

### 3.1. Type of Patient Access Barriers to Biologic Treatments in CEE and CIS

**Figure 1:**
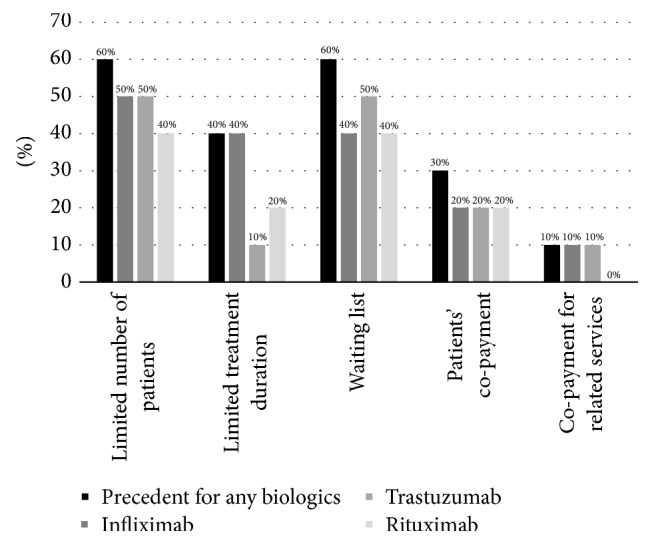
Frequency of reported patient access barriers to biologic medicines in participant countries (*n* = 10).

Figure 1 summarizes reported barriers of timely patient access for reimbursed biologic medicines in participant countries. Respondents had the opportunity to select multiple options. Volume limits on the number of patients who can be treated with public reimbursement, related waiting lists, and limited duration of reimbursed biologic treatment were reported as key barriers to patient access to biologics. It is noteworthy that waiting lists were reported even in haematology indications for rituximab and oncology indications for trastuzumab in some countries. Additionally, other barriers were mentioned: nonreimbursement of complementary diagnostics, additional eligibility criteria, or restricted reimbursement compared to the registered indication (e.g., reimbursement only for metastatic disease).

### 3.2. Can Uptake of Biosimilars Lower Hurdles to Patient Access for Biologic Medicines?

**Figure 2:**
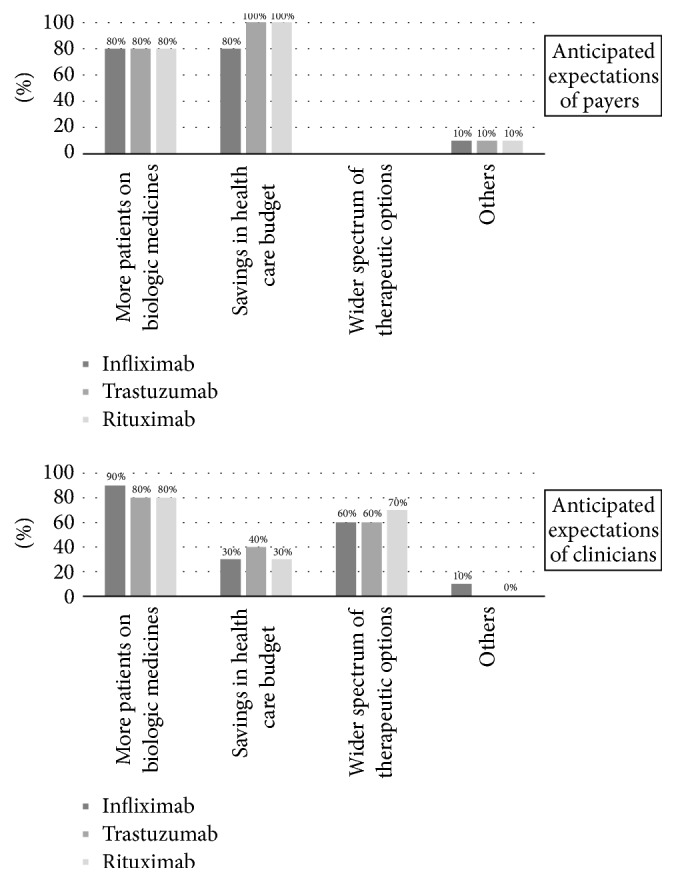
Expected benefits from treating more patients with biosimilars (*n* = 10).

Generally, the cost-saving potential and the capacity to improve patient access to reimbursed biologic medicines were mentioned as key benefits of increased utilisation of biosimilars. According to respondents' opinions, both clinicians and payers expect that treating more patients with reimbursed biologic medicines (i.e., improved patient access to biologics) would be the primary benefit of switching patients to biosimilars (Figure 2). The cost-saving potential of biosimilars for payers and the increased number of treatment options for clinicians were mentioned as secondary benefits. In the “other” option, some respondents mentioned that even the price of original products may be reduced due to price erosion generated by internal price referencing. However, other respondents expressed their concern that the generated cost savings may be invested in other sectors of health care or even outside the health care sector and thus not improve patient access.

### 3.3. Key Factors of Limited Patient Access to Biosimilars

#### 3.3.1. Indication Extrapolation

According to this survey, in general, payers in CEE/CIS countries seem to have no concerns with extrapolation to indications. EMA registration is well accepted even if pivotal phase III data were to be extrapolated to other indications without additional phase III clinical trial evidence. In contrast, respondents in some countries—for example, in Bulgaria and in Slovakia—expected negligible or restricted use of biosimilars by clinicians in indications with only extrapolated data. This is especially true in the field of oncology, where respondents anticipated concerns from clinicians about extrapolating biosimilar access to other indications.

#### 3.3.2. Consequences of Switching Patients to Biosimilars

**Figure 3:**
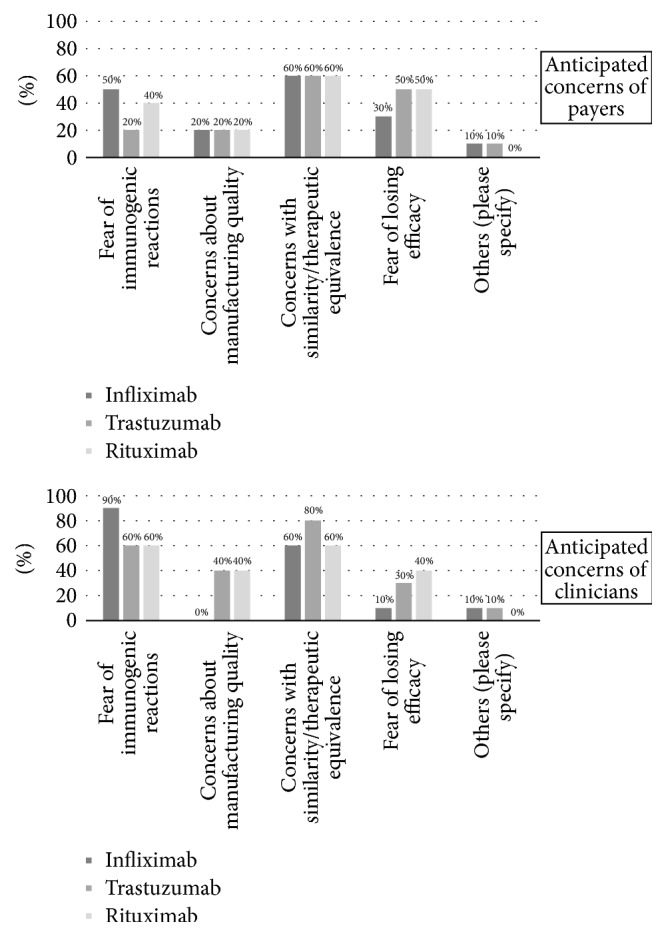
Concerns related to switching to biosimilars (*n* = 10).

According to respondents, immunogenicity after switching the patients to biosimilar medicines could be a major concern for clinicians, while limited information on therapeutic equivalence with their originator could be problematic for both clinicians and payers (see Figure 3). Interestingly, manufacturing quality was reported as a concern only in countries outside the European Union. Fear of political scandal as a consequence of any adverse outcomes related to switching was reported under “other” category.

### 3.4. Clinical Practice Related to Switching

In majority of the countries, switching from original biologic medicine to its biosimilar alternative is allowed. However, patients on maintenance biologic treatment were expected to stay on the original product. While majority of the countries apply tenders for public procurement of biologic medicines, the tender-winning biosimilars were expected to be used for both treatment-naïve patients and patients already on maintenance biologics only in Lithuania, Poland, and Slovakia. In Hungary, there are two separate tenders, one for treatment-naïve patients and one for patients already on maintenance biologics. In all other countries, the tender winner product should be prescribed only for the nontreated patients, while treated patients may continue their previous biologic treatment: switching to a more affordable biosimilar alternative is not mandatory.

### 3.5. Framework for Postmarketing Data Collection

Postmarketing data collection related to medicines in general was reported in all participant countries either in patient registries or based on payers' databases. Such activities are coordinated by the health insurance fund in Hungary, Poland, and Slovakia. Because of the abovementioned concerns related to biosimilars, all countries except Russia reported that pharmacovigilance data on biosimilars were regularly assessed or planned to be assessed in the near future according to EMA requirements. Additionally, national regulatory authorities in Hungary and Slovakia were reported to reassess data on effectiveness after registering a new biosimilar to ensure equal health gain with the originator product.

## 4. Discussion

While a few publications are available on biosimilar policies in higher income countries [8, 30–32], the information is even more limited from CEE/CIS countries [33]. Our study indicates significant access restrictions to original biologic medicines in CEE/CIS countries. Similar findings were presented in a recent publication on disease-modifying biologic antirheumatic medicines in 37 European countries, including Russia and Turkey [10]. In our policy survey, real-world experience for both the original and the biosimilar products was available only for infliximab. Interestingly, the reported restrictions in patient access were similar for original and biosimilar infliximab. No improvement in the patient access after biosimilar entry is an indicator of ineffective biosimilar policies in CEE/CIS countries. The main objective of biosimilar policy in countries with resource constrains should be to improve health gain at the population level by reducing barriers of patient access to reimbursed biologic medicines—both originator and biosimilar [34]. Expected barriers in patient access to biosimilar trastuzumab and rituximab were reported to be similar to current barriers to original trastuzumab and rituximab, as respondents did not expect significant changes in biosimilar policies. This finding calls for action and reconsideration of current policies in order to facilitate the use of biosimilar medicines in CEE/CIS countries [8].

Given reported access restrictions, it is not surprising that increasing the number of patients receiving reimbursed biologic treatments was considered the primary benefit of switching patients to biosimilar products in almost all participant countries. Value-based purchasing strategies require strong government interventions: biosimilars will not become the preferred option in clinical practice if biosimilar policies are ineffective and central government administration plays a passive role [35]. As with generic products, more affordable biosimilars should be the first-line options for the majority of patients [34]. This policy has been already implemented for biosimilars in treatment-naïve patients, for example, in one Italian region and Poland, Denmark, and Norway [8, 36–38].

Because infliximab, trastuzumab, and rituximab are mainly used as long-term maintenance therapies in chronic diseases, the societal benefits of access to more affordable biosimilars can be maximised by both starting all de novo patients on biosimilars and, as much as is clinically justifiable, switching patients to biosimilars [9]. Although there is still no supportive evidence related to the multiple switches of patients between biosimilar alternatives, the single switch of patients on original biologics to a biosimilar alternative under medical supervision should be mandated or incentivized [24]. This recommendation is in line with results of recent systematic reviews demonstrating no increased risk or loss of efficacy due to switching from original biologics to a biosimilar alternative [39–41]. Such practice is actively encouraged in Poland [42].

As a general practice, our survey revealed that switching to biosimilar products is allowed in many countries and may be initiated at the discretion of the physician. This passive policy was confirmed in a recent policy survey in CEE countries [33]. In contrast, in Lithuania, Poland, and Slovakia, where tender-winning products are likely to be used for patients on maintenance original biologic therapy, mandatory switching to biosimilars is already partially implemented [43]. If tenders are nonexclusives—that is, both the original biologic medicine and its biosimilar version(s) are included in the formulary—physicians can choose which biologic should be prescribed for a patient: this represents a more passive biosimilar policy and potentially slower uptake of biosimilars [8]. Introduction of prescription quotas may also facilitate the uptake of biosimilars. In Hungary, for example, physicians are incentivized to prescribe the preferred biologics in a minimum 40% of their current patients [33]. A similar, but less ambitious, approach was reported from Belgium: as a consequence of a multistakeholder agreement, biosimilars should be used in at least 20% of new patients [44].

Uncertainty related to switching may be handled by risk-management strategies [9]. Ex post risk-management strategy mandates postmarketing data collection and a risk-management plan for manufacturers of biosimilar products and/or health care institutions. Our results indicate that a conceptual framework for a strengthened pharmacovigilance system for biosimilars based on additional real-world data collection exists in all countries. France is a good example for the significance of such data collection: based on real-world evidence, the French drug agency changed its initially negative attitude towards switching and stated that switching patients to biosimilars under medical supervision should not be discouraged [45]. According to a recent survey conducted in New Zealand, most physicians indicated that they would prescribe biosimilars for clinical conditions in which biosimilars are cost-effective alternatives to original biologic medicines. However, they also highlighted the necessity of communication guidance for clinicians on how to explain biosimilars effectively to patients in order to reduce potential objections [46].

A recent paper summarizes potential government interventions to facilitate the utilisation of biosimilars in lower income countries (see Table 3) [9].

Generalizability of our findings is limited, as our policy research engaged only two experts (representing both public and private sectors) in each participant country. Although survey respondents had the opportunity to consult with additional local experts, their opinion may not be fully representative for the entire country. However, as our findings are in line with conclusions of other publications, the trends presented in this study may provide relevant input to reconsideration of current biosimilar policies in CEE/CIS countries.

## 5. Conclusion

Our survey indicated a significant unmet need for more affordable biologic therapies in the CEE/CIS countries. The key concerns about the increased utilisation of biosimilars were limited evidence on therapeutic equivalence and expected adverse immunologic reactions. Nevertheless, the role of biosimilars in increasing patient access to reimbursed biologics was acknowledged by almost all experts. Policy-makers have to take a strategic approach to increase societal benefit from biosimilar medicines. Relying on free-market incentives may not be strong enough; hence, active government interventions instead of “passive disinvestment” policies are needed to correct for current access limitations.

## Figures and Tables

**Table 1:** Affiliation of survey respondents.

Country	Public sector			Private sector
	Academic policy expert	HTA expert	Payer/reimbursement committee	Industry
Bulgaria	X			X
Czech Republic	X			X
Hungary			X	X
Kazakhstan		X		X
Latvia			X	X
Lithuania	X			X
Poland			X	X
Romania	X			X
Russia		X		X
Slovakia			X	X

**Table 2:** Questions of the reimbursement policy survey.

*General questions related to all biologics and specific biologic therapies*	
(Q1) Please select which type of access limit is applied on the patient population in the therapeutic use of original biologics/biosimilars^*∗*^ in your country! (multiple choice)	(A) Access limit on the number of treated patients – i.e. patients above the volume limit cannot be treated with biologics
	(B) Access limit on treatment duration/cycles – i.e. treatment duration/cycles is maximised by payers
	(C) Waiting lists for eligible patients – i.e. timely access to biologics is not guaranteed for patients
	(D) Patient co-payment for biologics – i.e. high co-payment limits patient access
	(E) Patient co-payment for related services – i.e. high co-payment limits patient access to necessary diagnostic services
	(F) Limited institutional access – e.g. biologics can be prescribed only in limited number of specialist centers
	(G) Other (please specify)

(Q2) What are the main (or expected) benefits of a clinician/payer from switching patients treated with original biologics to a biosimilar^*∗*^ product? (multiple choice)	(A) More patients are treated with biologics
	(B) Savings in health care budget
	(C) Wider spectrum of treatment options
	(D) Other (please specify)

(Q3) Is indication extrapolation accepted in your country by payers/clinicians for biosimilars^*∗*^? (single choice)	(A) Yes + Answers in details
	(B) No + Answers in details

(Q4) What are the main concerns (expected concerns) of a clinician/payer of switching patients treated with original biologics to biosimilars^*∗*^ in your country? (multiple choice)	(A) Fear of immunogenic reactions
	(B) Concerns about manufacturing quality
	(C) Concerns with similarity/therapeutic equivalence
	(D) Fear of losing efficacy
	(E) Other (please specify)

*Specific questions related to experiences with biosimilar infliximab*	
(Q5) Please provide information about switching patients from original infliximab to its biosimilar alternative in your country! (single choice)	(A) Switching patients on maintenance original infliximab treatment is not allowed
	(B) Switching patients on maintenance original infliximab treatment is allowed
	(C) Switching patients on maintenance original infliximab treatment is incentivized
	(D) Switching patients on maintenance original infliximab treatment is mandatory

(Q6) How switching to biosimilar infliximab is implemented for patients on maintenance original infliximab treatment? (single choice)	(A) Patients on maintenance original infliximab treatment are switched to the cheapest biosimilar alternative and stay on the same infliximab product (i.e. single switch)
	(B) Patients on maintenance original infliximab treatment are always switched to another biosimilar infliximab product when a cheaper biosimilar alternative becomes available (i.e. multiple switch)
	(C) Patients on maintenance original infliximab treatment stay on the original infliximab product even after the availability of cheaper biosimilar alternatives (no switching)
	(D) Other (please specify)

(Q7) Is tendering system applied for purchasing biosimilar infliximab for specific patient groups (e.g. Rheumatoid Arthritis, Psoriatic Arthritis, Colitis Ulcerosa, Crohn's Disease patients)? (multiple choice)	(A) Tendering system to purchase biosimilar infliximab is not applied
	(B) Centralized tendering system is applied at national level i.e. one purchasing body coordinates the tendering
	(C) Decentralized tendering system is applied at regional level with national coordination i.e. tendering rules are set nationally, but implemented regionally
	(D) Other (please specify)

*General questions related to all biologics and specific biologic therapies*	
(Q8) Does your country apply incentives to generate real world evidence related to the use biosimilar products? e.g. quality of life data, survival data etc. (multiple choice)	(A) No, such incentives are not applied
	(B) Yes, incentives are applied for collecting real world evidence at national level
	(C) Yes, incentives are applied for collecting real world evidence through international collaborations
	(D) Other (please specify)

(Q9) What are the potential data sources for real world evidence of biosimilars^*∗*^ in your country? (multiple choice)	(A) Patient registries
	(B) Payers' database
	(C) Other (please specify)

(Q10) Is the safety profile and the real world effectiveness of biosimilars^*∗*^ assessed or evaluated after the registration? (multiple choice)	(A) No
	(B) Yes, safety profile is assessed
	(C) Yes, effectiveness is evaluated
	(D) Yes, cost-effectiveness is evaluated
	(E) Other (please specify)

^*∗*^In different spreadsheets, original biologics were replaced with original infliximab, trastuzumab, or rituximab; biosimilars were replaced with biosimilar infliximab, trastuzumab, or rituximab.

**Table 3:** Potential policy actions to maximise the societal benefit of biosimilars [9].

Areas for intervention	Potential policy actions
Public administration of biosimilar medicines	Expedited price and reimbursement process to facilitate the timely market entry of biosimilars
	Introduction of administrative tools and policy measures to incentivize the choice for more affordable biosimilars

Clinical guidelines	Multisource biologic medicines should be first-line biologic therapy for all patients. More expensive patented biologic medicines with no proven significant clinical benefit compared to biosimilar medicines should be only second line options
	Single switch of patients from an original biologic medicine to its more affordable biosimilar alternative under medical supervision should be mandated after patent expiry
	Physicians should not only be informed about scientific evidence on biosimilars but also guided on how to educate appropriately their patients on these medicines

Evidence base of policy decisions	Cost-effectiveness or cost-utility analysis is applied to judge the full economic value of biosimilar medicines except in those cases, when biosimilar medicines are compared to their original biologic alternative for treatment-naïve patients.
	Budget impact analysis is applied to estimate (1) the savings from biosimilar medicines, if there is no patient access limit to biologic medicines, or (2) the incremental budget, if patient access to biologic medicines is restricted

Management of uncertainty related to policy decisions	Ex ante risk management: calculation of threshold for the risk of immunogenicity, where not switching patients to biosimilar medicines is the preferred option from the payers' perspective
	Ex post risk management: mandate of vigorous pharmacovigilance data collection and risk-management plan in case of increased risk of immunogenicity. The risk-management plan may even include risk-sharing agreements with manufacturers of biosimilar medicines
